# Changes in retinal and choriocapillaris density in diabetic patients receiving anti-vascular endothelial growth factor treatment using optical coherence tomography angiography

**DOI:** 10.1186/s40942-019-0192-9

**Published:** 2019-12-10

**Authors:** Felipe F. Conti, Weilin Song, Eduardo B. Rodrigues, Rishi P. Singh

**Affiliations:** 1 Cole Eye Institute, Cleveland Clinic Foundation, 2022 E. 105th St, i Building, Cleveland, OH 44106 USA; 2 Cleveland Clinic Lerner College of Medicine, 9500 Euclid Avenue, NA21, Cleveland, OH 44195 USA; 3 Center for Ophthalmic Bioinformatics, Cole Eye Institute, Cleveland Clinic, 10685 Carnegie Ave, Cleveland, OH 44106 USA; 4 Federal University of São Paulo, 781 Pedro de Toledo Street, São Paulo, SP 04039-032 Brazil

**Keywords:** Anti-vascular endothelial growth factor, Capillary perfusion density, Diabetic retinopathy, Macular edema, Optical coherence tomography angiography

## Abstract

**Background:**

Optical coherence tomography angiography (OCTA) enables detailed, non-invasive assessment of ocular vasculature. This study uses OCTA imaging to evaluate choriocapillaris and retinal capillary perfusion density (CPD) changes in diabetic retinopathy following anti-vascular endothelial growth factor (VEGF) treatment.

**Methods:**

Records of 38 eyes at a single institution were reviewed, grouped as non-diabetic controls (19 eyes), diabetes mellitus patients with diabetic retinopathy (DR, 19 eyes) and macular edema (DME). DR eyes were imaged at baseline, 6-months and 12-months after anti-VEGF treatment. Quantitative analyses assessed CPD of the choriocapillaris and retinal plexus.

**Results:**

DR eyes showed decreased choriocapillaris whole-image CPD (62.6 ± 6.1 vs. 68.4 ± 5.1, p < 0.003), foveal CPD (61.2 ± 7.4 vs. 66.3 ± 9.8, p < 0.014), and parafoveal CPD (61.9 ± 6.6 vs. 68.2 ± 4.8, p < 0.002) at baseline. DR eyes also showed decreased retinal density, including whole-image CPD (46.9 ± 5.1 vs. 50.7 ± 5.6, p < 0.04), foveal CPD (27.6 ± 5.9 vs. 34.1 ± 6.1, p < 0.002), and parafoveal CPD (49.0 ± 5.6 vs. 53.1 ± 6.0, p < 0.011). Following 12 months of anti-VEGF treatment, no changes to retinal or choriocapillaris or CPD were observed. Retinal central subfield thickness decreased (397.1 ± 93.2 µm vs. 294.2 ± 71.5 µm, p < 0.005). Lastly, FAZ area (0.307 ± 0.133 mm^2^ vs. 0.184 ± 0.058 mm^2^, p = 0.008) and perimeter (2.415 ± 0.692 mm^2^ vs. 1.753 ± 0.408 mm^2^, p = 0.002) were increased in DR eyes at baseline. No changes to FAZ area or perimeter were seen with anti-VEGF treatment in DR eyes.

**Conclusions:**

Compared to control, choriocapillaris and retinal CPD are reduced in DR, while FAZ area and perimeter are increased. No retinal capillary or choriocapillaris CPD changes were observed in DR eyes following anti-VEGF treatment.

## Background

Diabetes mellitus (DM) affects over 200 million people world-wide [1], including 30.3 million in the United States alone [2]. Diabetic retinopathy (DR) is present in 28.5% of the diabetic population and has been a major concern among visual care professionals [3]. In the effort to control this disease, anti-vascular endothelial growth factor (anti-VEGF) has been successfully used to treat diabetic macular edema (DME) and proliferative diabetic retinopathy (PDR) resulting in improved visual and anatomic outcomes [4].

Vascular abnormalities related to hyperglycemic states have been documented in other eye tissues. Loss of choriocapillaris, tortuous blood vessels, microaneurysms, drusenoid deposits on Bruch’s membrane, and choroidal neovascularization have been observed in the choroid of diabetic patients [5–7]. However, little is known about the effect of DR treatment in the choroid. Lee et al. in a prospective study of 101 patients, examined the choroidal thickness (CT) changes after anti-VEGF injection, PRP or both for diabetic retinopathy treatment. They suggested that choroidal thickness decreased after all treatments [8]. Laíns et al. in a cross-sectional study of 50 eyes, analyzed the effect of anti-VEGF in CT of DR patients, reporting similar results of thickness reduction [9]. Yiu et al. in a retrospective cohort of 59 eyes, examined the effect of anti- VEGF in eyes with DME and also reported central CT decrease after therapy. Nevertheless, all of these analyses were made with optical coherence tomography (OCT) images without any flow assessment.

OCT angiography is a new imaging modality that safely, quickly, and noninvasively assesses the microvascular changes from diabetic ocular disease. Several studies have used this technology to investigate the effects of anti-VEGF treatment on retinal vessels [10, 11]. Gill et al. in a retrospective, observational case series of 20 eyes, reported a significant foveal avascular zone (FAZ) reduction over time [12]. Khalil et al. in a pilot study of 15 DME patients, reported no statistical difference in retinal capillary density and FAZ after a single anti-VEGF injection [13]. However, these studies are confined to the retinal boundaries. Herein, this study is proposed to assess retinal and choriocapillaris perfusion changes in DM patients receiving anti-VEGF treatment using OCTA.

## Methods

### Study design

This cohort study was performed at Cole Eye Institute, Cleveland, OH after receiving approval from the Cleveland Clinic Investigational Review Board (IRB). A comprehensive chart review was performed to assess ophthalmic data. All study related procedures were performed in accordance with good clinical practice (International Conference on Harmonization of Technical Requirements of Pharmaceuticals for Human Use [ICH] E6), applicable FDA regulations, and the Health Insurance Portability and Accountability Act. The primary objective of this study was to assess choriocapillaris and retinal capillary perfusion density (CPD) changes in diabetic retinopathy patients treated with anti-VEGF using OCTA.

### Participants and image acquisition

Patients in the diabetic group were part of the SWAP-TWO prospective, interventional study, which examined the efficacy of a fixed dosing regimen of intravitreal aflibercept on diabetic macular edema (DME) [14]. In the SWAP-TWO trial, eligible participants were aged ≥ 18 years, presented with foveal-involving retinal edema secondary to diabetic retinopathy (DR), and initiated anti-VEGF therapy for DR treatment. Patients were treated with intravitreal aflibercept injections (IAI) administered monthly until OCT demonstrated no evidence of fluid, followed by mandatory IAI once every 2 months. OCT imaging was performed at the baseline, 6 and 12 month visits. Detailed review of the protocol and study design are included within the SWAP-TWO 6-month interim report [14]. History of previous anti-VEGF injections was not a criterion for exclusion. The control group consisted of patients without DR or any signs of retinal disease, as confirmed with biomicroscopy records.

**Fig. 1:**
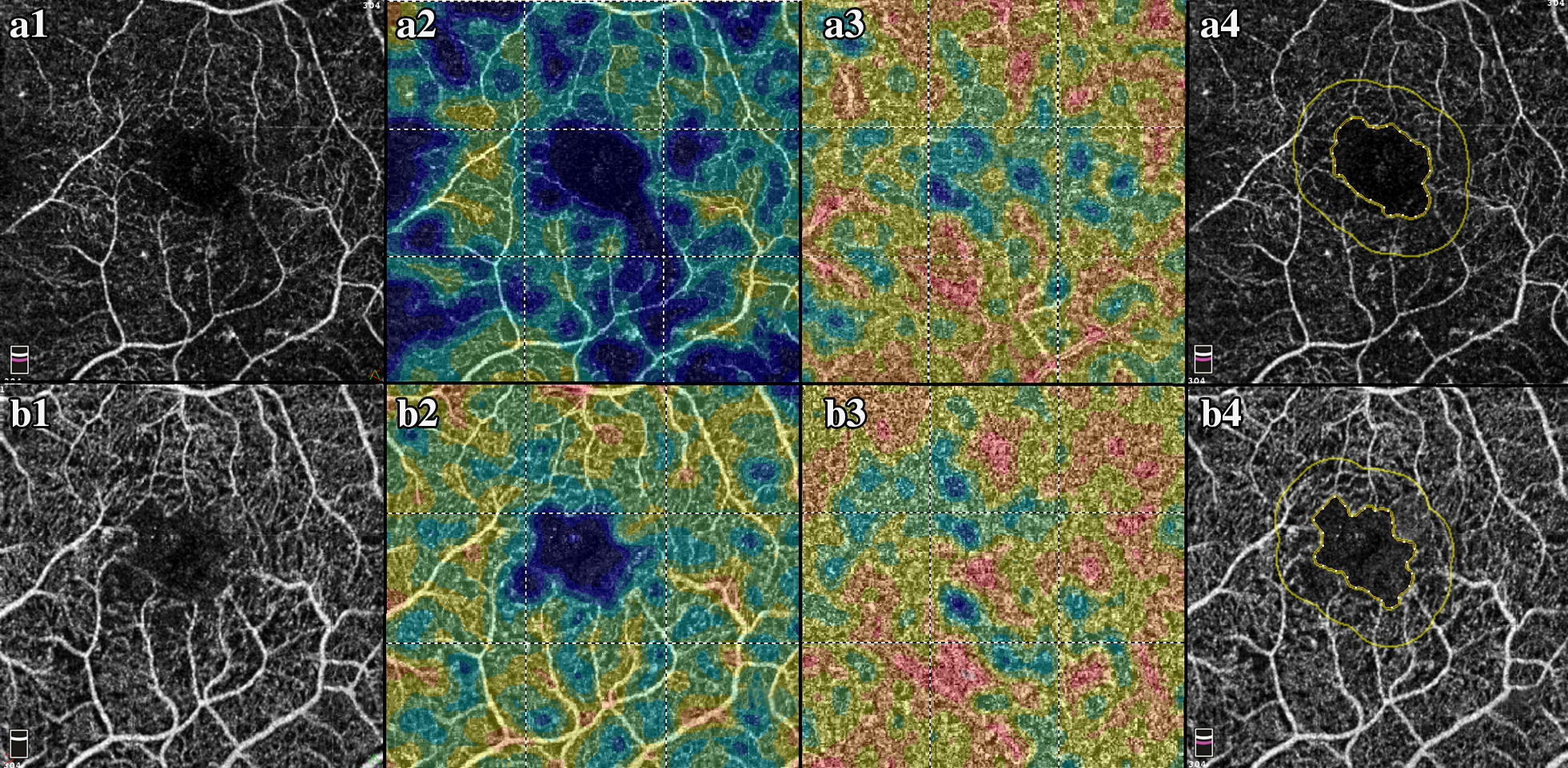
Optical coherence tomography angiography and capillary perfusion density analysis. **a1** Example of En Face OCT of a diabetic retinopathy patient at baseline. **a2** Full retina capillary perfusion density analysis. **a3** Choriocapillaris perfusion density analysis. **a4** FAZ area analysis. **b1** Example of En Face OCT of a diabetic retinopathy patient after 12 month of anti-VEGF treatment. **b2** Full retina capillary perfusion density analysis. **b3** Choriocapillaris perfusion density analysis. **b4** FAZ area analysis

Patients were excluded from the study if (1) the presence of any other vitreoretinal diseases that may affect ocular circulation (including retinal vascular occlusion, central serous retinopathy or macular dystrophies) was identified; (2) the presence of low quality scans which hindered CPD analysis (i.e.: low signal strength, gaze instability, or media opacity) was noticed. Variables such as DR severity (mild, moderate, severe NPDR, and PDR), number of previous anti-VEGF injections, and best corrected visual acuity (BCVA) were collected for each patient in the diabetic group. A single eye of each patient was included. Eyes were imaged with the Optovue Avanti RTVue XR spectral-domain OCT (SD-OCT) (Fig. 1) from January 2015 to January 2018. The specifics of the scanning protocol have been previously described [15]. Quantitative CPD analysis was performed with ReVue software version 2017.1.0.129 (Optovue, Inc, Fremont, CA). The built-in software calculates the 6 × 6 mm whole-image CPD by computing the percentage area occupied by detected OCTA vasculature. Vessels’ pixels area is ‘true’ while background/noise is ‘false’. This binary mask is then used to generate a 2D local density map and automatically calculated density values in grid sectors. Auto-segmentation was used to define anatomical borders for CPD analysis and for FAZ area demarcation. Foveal CPD was calculated using a central circle with a diameter of 1 mm, while parafoveal CPD was calculated using an inner diameter of 1 mm and outer diameter of 3 mm. Manual corrections were made if any scan errors were identified.

### Statistical analysis

Categorical variables were described using frequencies and percentages, while continuous variables were described using means, standard deviations, and ranges. Relationships between categorical variables were assessed using Kruskal–Wallis tests (for ordered variables), while relationships between continuous variables were assessed using T-tests, one-way ANOVA test (for normally distributed variables), or Kruskal–Wallis tests (for non-normally distributed variables). Analyses were performed using SPSS Statistics^®^ software version 25 (Chicago, SPSS Inc.).

## Results

### Demographics

Thirty-eight age-matched patients had their charts reviewed, of which 19 were DR patients (15 NPDR and 4 PDR) and 19 were non-diabetic control patients. The DR patients were not anti-VEGF naïve, and previously received an average of 5.1 injections (± 0.9) and had an average washout time of 44.4 days (± 21.2). By 6 months, 13 (68%) patients still required monthly treatment and 6 (32%) received IAI every 2 months. By 12 months, 5 (26%) patients received monthly treatment while 14 (74%) patients received injections every 2 months. Patients underwent an average of 5.2 injections prior to switching to receiving injections every 2 months.

### Baseline choroidal and retinal vascular indices and thickness values

Baseline comparison of choroidal and retinal vascular indices and thickness between the control group and DM patients under initiation of anti-VEGF treatment are shown in Table 1. Eyes with DR had significantly decreased choriocapillaris density. Compared to controls, patients with DR showed decreased whole-image CPD (62.6 ± 6.1 vs. 68.4 ± 5.1, p < 0.003), foveal CPD (61.2 ± 7.4 vs. 66.3 ± 9.8, p < 0.014), and parafoveal CPD (61.9 ± 6.6 vs. 68.2 ± 4.8, p < 0.002) at baseline. DR eyes also showed significantly decreased retinal density in comparison to control eyes, including whole-image CPD (46.9 ± 5.1 vs. 50.7 ± 5.6, p < 0.04), foveal CPD (27.6 ± 5.9 vs. 34.1 ± 6.1, p < 0.002), and parafoveal CPD (49.0 ± 5.6 vs. 53.1 ± 6.0, p < 0.011). There was no significant difference in choroidal thickness between the two groups, but DR patients had significantly increased retinal central subfield thickness (CST) (397.1 ± 93.2 µm vs. 276.6 ± 22.9 µm, p < 0.0001) compared to control patients.

**Table 1:** Baseline comparison between control group and diabetic retinopathy patients under initiation of anti-VEGF treatment

	Control group (n = 19)	Diabetic retinopathy (n = 19)	*p*-value
Age (SD)	64.4 (± 9.7)	63.5 (± 9.3)	0.7^∆^
FAZ area mm^2^	0.184 (± 0.058)	0.307 (± 0.133)	0.008*
Perimeter mm^2^	1.753 (± 0.408)	2.415 (± 0.692)	0.002*
Choroid thickness µm	317.8 (± 15.3)	335.9 (± 39.1)	0.07^∆^
Choriocapillaris whole image density %	68.4 (± 5.1)	62.6 (± 6.1)	*0.003* ^∆^
Choriocapillaris foveal area density %	66.3 (± 9.8)	61.2 (± 7.4)	0.014*
Choriocapillaris parafoveal area density %	68.2 (± 4.8)	61.9 (± 6.6)	*0.002* ^∆^
Retinal whole image density %	50.7 (± 5.6)	46.9 (± 5.1)	*0.04* ^∆^
Retinal foveal area density %	34.1 (± 6.1)	27.6 (± 5.9)	*0.002* ^∆^
Retinal parafoveal area density %	53.1 (± 6.0)	49.0 (± 5.6)	0.011*
CST µm	276.6 (± 22.9)	397.1 (± 93.2)	*<* 0.0001*
Visual acuity ETDRS	81.3 (± 4.6)	69.7 (± 8.1)	*<* 0.0001*

Italic values indicate the statistical significance

*SD* standard deviation, *FAZ* foveal avascular zone, *CST* central subfield thickness, *ETDRS* early treatment diabetic retinopathy study

^∆^Unpaired T-test, * Mann–Whitney test

Baseline foveal avascular zone (FAZ) area and perimeter values are displayed in Table 1. In comparison to the control group, eyes with DR showed significantly increased FAZ area (0.300 ± 0.133 mm^2^ vs. 0.184 ± 0.058 mm^2^, p < 0.008) and perimeter (2.415 ± 0.692 mm^2^ vs. 1.753 ± 0.408 mm^2^, p < 0.002).

### Choroidal and retinal vascular indices and thickness following anti-VEGF treatment

**Fig. 2:**
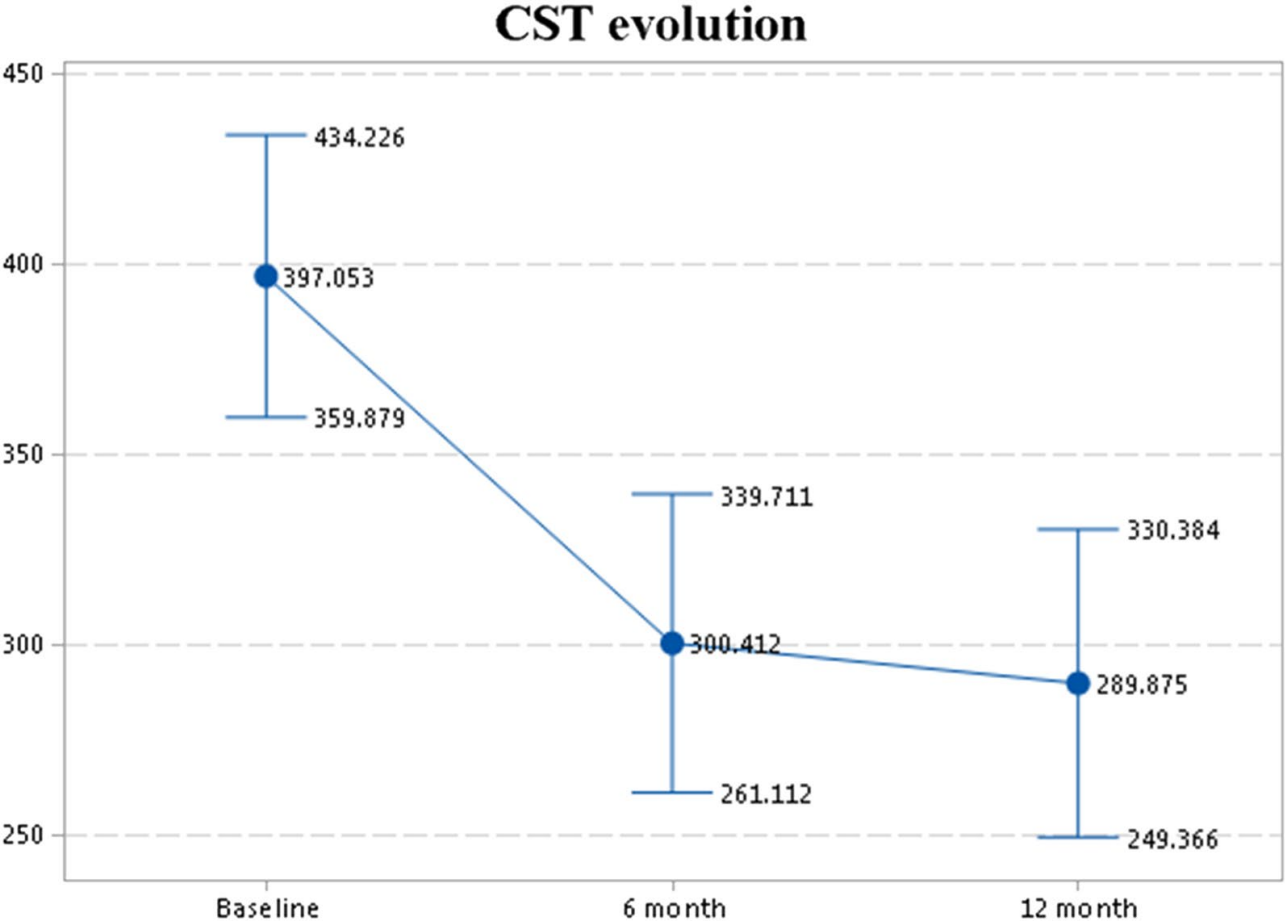
Central subfield thickness evolution during anti-VEGF treatment

Comparison between baseline, 6-month, and 12-month time point measurements after anti-VEGF treatment for DR patients are shown in Table 2. Following treatment, eyes with DR did not show significant changes in choriocapillaris whole-image CPD, foveal CPD, or parafoveal CPD. There was also no significant overall change in retinal whole-image CPD, foveal CPD, or parafoveal CPD between baseline, 6-month, and 12-month time points. No change in choroid thickness was seen between the three time points, but retinal CST decreased between baseline and 6-months (300.4 ± 67.8 vs. 397.1 ± 93.2, p < 0.001) (Fig. 2). FAZ area and perimeter values for the three time points (baseline, 6-month, and 12-month) are displayed in Table 2. No difference was seen in the FAZ area or perimeter between baseline, 6-month and 12-month time points after anti-VEGF treatment.

**Table 2:** Comparison between baseline, 6 month and 12 month time-point measurements after anti-VEGF treatment

	Baseline (n = 19)	6 month (n = 17)	12 month (n = 16)	Overall comparison p-value	Baseline-6 month p-value	6 month–12 month p-value
FAZ area mm^2^ (SD)	0.307 (± 0.133)	0.3 (± 0.1)	0.313 (± 0.1)	0.6^†^	0.5*	0.10*
Perimeter mm^2^ (SD)	2.415 (± 0.692)	2.4 (± 0.5)	2.394 (± 0.4)	0.7^†^	0.9*	0.3*
Choroid thickness µm (SD)	335.9 (± 39.1)	347.2 (3 ± 8.1)	336.8 (± 44.6)	0.7^†^	0.4*	0.3^∆^
Choriocapillaris whole image density % (SD)	62.6 (± 6.1)	63.4 (± 6.4)	63.9 (± 6.9)	0.9^†^	0.7*	0.5*
Choriocapillaris foveal area density % (SD)	61.2 (± 7.4)	60.6 (± 8.8)	59.1 (± 7.9)	0.85^†^	0.8^∆^	0.3*
Choriocapillaris parafoveal area density % (SD)	61.9 (± 6.6)	63.2 (± 6.6)	64.1 (± 7.3)	0.9^†^	0.5*	0.7*
Retinal whole image density % (SD)	46.9 (± 5.1)	45.3 (± 5.2)	45.7 (± 5.0)	0.5^†^	0.4*	0.5*
Retinal foveal area density % (SD)	27.6 (± 5.9)	25.2 (± 6.9)	25.0 (± 6.8)	0.2^†^	0.3*	0.07*
Retinal parafoveal area density % (SD)	49.0 (± 5.6)	47.5 (± 5.7)	48 (± 4.9)	0.6^†^	0.1^∆^	0.6*
CST µm (SD)	397.1 (± 93.2)	300.4 (± 67.8)	294.2 (± 71.5)	*0.005* ^†^	0.001*	0.7*
Visual acuity ETDRS (SD)	69.6 (± 7.9)	72.2 (± 7.9)	73.9 (± 7.2)	0.3^†^	0.2*	0.3*

Italic values indicate the statistical significance

*SD* standard deviation, *FAZ* foveal avascular zone, *CST* central subfield thickness, *ETDRS* early treatment diabetic retinopathy study

* T-test, ^∆^Mann–Whitney, ^†^One-way ANOVA

### Visual acuity

At baseline, DR patients had a significantly lower ETDRS score than control patients (69.7 ± 8.1,  ≅ 20/40 vs. 81.3 ± 4.6, ≅ 20/25 p < 0.0001). There was no significant improvement in visual acuity following 1 year of treatment with anti-VEGF therapy (69.7 ± 8.1, ≅ 20/40 vs. 73.9 ± 7.2, ≅ 20/32; p = 0.3).

## Discussion

Results from this study examining retinal and choriocapillaris perfusion changes in DM patients receiving anti-VEGF treatment suggest that while eyes with DR have decreased choroidal and retinal vascular density compared to control eyes, anti-VEGF treatment does not significantly alter the choroidal or retinal vascular density. Given that there is limited current scientific knowledge about choriocapillaris alterations during diabetic retinopathy, this study adds important information about changes in choroidal thickness and vascular indices in eyes with DR at baseline and following anti-VEGF treatment.

Choroidal thickness measurements in eyes with DME have been inconsistent, with studies suggesting diverging reports of choroidal thickening [16], thinning [17], and no change [18]. One explanation for these differing results is the significant variability of factors across retrospective, cross-sectional studies. Choroidal thickness has been shown to vary with age, refractive error, and even time of measurement [19]. Additionally, anti-VEGF therapy has been shown to cause choroidal thinning in other ocular diseases such as age-related macular degeneration (AMD) [20]. In this study, DR patients at baseline did not have a significant difference in choroidal thickness when compared to the non-diabetic control group, although there was a positive trend that may become significant in larger cohorts. Choroidal thickness also did not significantly change in eyes with DR following 1 year of anti-VEGF therapy. Yiu et al. in a retrospective cohort of 59 eyes, showed that anti-VEGF naïve DME patients had decreased choroidal thickness after an average of 2.73 injections over 6 months [19]. Although the findings appear to conflict with the results reported here, one explanation could be that the patients in this study were not naïve to anti-VEGF treatment. While there may initially be choroidal thinning with anti-VEGF treatment, our results suggest that these changes do not progress with long-term treatment.

Choi et al. in a cross-sectional study of 152 eyes, used OCTA to examine retinal and choriocapillaris microvasculature and found abnormalities in all stages of diabetic retinopathy [21]. Functional imaging studies also showed a reduction in choroidal blood flow in eyes with diabetic retinopathy [22]. Additionally, choriocapillaris density was shown to be decreased in DR compared to control patients, suggesting that choroidal vasculature alterations could be contributing to the pathogenesis of diabetic eye disease [23]. The present study is in agreement with current knowledge that DR patients had significantly decreased choriocapillaris density compared to the control group. However, no significant change was noticed in choriocapillaris density after 1 year of anti-VEGF treatment. One possibility is that while the integrity of the choroidal vasculature is altered in diabetic retinopathy, the mechanism by which anti-VEGF therapy decreases retinal edema and improves visual acuity does not involve reversing the alterations to choroidal vasculature.

FAZ area was shown to be increased in DR eyes compared to control eyes in both area and perimeter, which supports findings by previous studies [24, 25]. Additionally, this study reports no change in FAZ area after 1-year treatment with intravitreal anti-VEGF. The results of other studies reporting the effects of chronic anti-VEGF therapy on FAZ area are conflicting. While some reports have stated that repeated intravitreal anti-VEGF injections can reduce the progression of the ischemic injury in patients with retinal microangiopathies [12, 26], others have not noticed any changes [11, 13], and others have even worsening of the capillary drop out [27–30]. Since none of the papers which question the protective role of anti-VEGF in ophthalmic ischemic injury enroll exclusively treatment-naïve patients, it can be hypothesized that, even if anti-VEGF does assist in reducing the ischemic injury in the foveal area, this outcome might reach a ceiling effect with continuous treatment. This hypothesis would explain why the present study fails to show FAZ area changes after chronic therapy.

Several clinical trials and smaller studies have demonstrated the significant visual acuity improvement of DME patients following anti-VEGF therapy [31, 32]. In our study, DR patients did not demonstrate significant improvement in visual acuity following anti-VEGF therapy. While baseline mean BCVA of 69.9 ETDRS letters (≅ 20/40) improved 4.3 letters after 12-months (p < 0.3) in the present study, baseline BCVA was 59.5 ETDRS letters (≅ 20/63) in the VISTA/VIVID trial anti-VEGF group, with an average improvement of 11.1 letters [33]. The high baseline BCVA could explain why a significant improvement in visual acuity following anti-VEGF treatment was not observed.

The significant decrease in CST after 6 months of anti-VEGF treatment is evidence that aflibercept effectively reduced retinal edema in patients with DR. However, the association between anatomical and functional outcomes cannot be drawn. The reduction of CST without accompanying improvements in visual acuity supports previous studies demonstrating only a modest correlation between OCT-measured center point thickness and visual acuity, suggesting that OCT measurements cannot reliably substitute as a surrogate for functional improvements [34, 35]. There was not a significant improvement in visual acuity during the study, however we believe this is due to the fact that mean BCVA was already high (± 70 letters) at baseline, leaving limited room for improvement.

Limitations of this study include its retrospective design, the small sample size, the absence of anti-VEGF naïve patients, the lack of patients with PDR and absence of DME, and the use of only one anti-VEGF agent (aflibercept). Strengths of this paper include the controlled dose and administration regimen of the anti-VEGF agent across patients, close follow-up, the use of the most sensitive method for assessing capillary density changes (the built-in AngioVue system) which enables image acquisition and quantification of results [36], and similar ages between control and DR groups. Although the presence of baseline edema may have influenced the readout of retinal and choriocapillaris results, this was less of a problem as edema resolved with treatment. Comparison between the same eyes at baseline and following anti-VEGF treatment also addresses many of the confounding variables associated with measurement of choroidal thickness and vascular indices.

## Conclusions

This study showed that OCTA is capable of detecting capillary perfusion changes in the retina and choriocapillaris of diabetic patients compared to healthy individuals, and is therefore a reliable technique for assessing microvasculature changes. The research findings also demonstrate that OCTA did not detect changes to choriocapillaris and retinal densities in DR patients with anti-VEGF treatment, although future randomized controlled trials with naïve patients, larger sample size, and extended observation periods might better evaluate perfusion changes after intravitreal treatment.

## Data Availability

The datasets used and/or analyzed during the current study are available from the corresponding author on reasonable request.

## References

[1] International Diabetes Federation. IDF Diabetes Atlas. 2015. 10.1289/image.ehp.v119.i03. 35914061

[2] McGinnis Sandra L., Zoske Frances M. (2008). The Emerging Role of Faith Community Nurses in Prevention and Management of Chronic Disease. Policy, Politics, & Nursing Practice.

[3] Zhang X, Saaddine JB, Chou C-F (2010). Prevalence of diabetic retinopathy in the United States, 2005–2008. JAMA.

[4] Stewart MW (2016). Treatment of diabetic retinopathy: recent advances and unresolved challenges. World J Diabetes..

[5] Lutty GA (2017). Diabetic choroidopathy. Vis Res.

[6] Hidayat AA, Fine BS (1985). Diabetic choroidopathy. Ophthalmology.

[7] Wang JC, Laíns I, Providência J (2017). Diabetic choroidopathy: choroidal vascular density and volume in diabetic retinopathy with swept-source optical coherence tomography. Am J Ophthalmol.

[8] Lee SH, Kim J, Chung H, Kim HC (2014). Changes of choroidal thickness after treatment for diabetic retinopathy. Curr Eye Res.

[9] Laíns I, Figueira J, Santos AR (2014). Choroidal thickness in diabetic retinopathy: the influence of antiangiogenic therapy. Retina..

[10] Lee J, Moon BG, Cho AR, Yoon YH (2016). Optical coherence tomography angiography of DME and its association with anti-VEGF treatment response. Ophthalmology.

[11] Michalska-Małecka K, Heinke Knudsen A (2017). Optical coherence tomography angiography in patients with diabetic retinopathy treated with anti-VEGF intravitreal injections. Medicine.

[12] Gill A, Cole ED, Novais EA (2017). Visualization of changes in the foveal avascular zone in both observed and treated diabetic macular edema using optical coherence tomography angiography. Int J Retina Vitreous.

[13] Ghasemi Falavarjani K, Iafe NA, Hubschman JP, Tsui I, Sadda SR, Sarraf D (2017). Optical coherence tomography angiography analysis of the foveal avascular zone and macular vessel density after anti-VEGF therapy in eyes with diabetic macular edema and retinal vein occlusion. Investig Ophthalmol Vis Sci..

[14] Babiuch AS, Conti TF, Conti FF (2019). Diabetic macular edema treated with intravitreal aflibercept injection after treatment with other anti-VEGF agents (SWAP-TWO study): 6-month interim analysis. Int J Retina Vitreous.

[15] Conti FF, Young JM, Silva FQ (2018). Repeatability of split-spectrum amplitude-decorrelation angiography to assess capillary perfusion density within optical coherence tomography. Ophthalmic Surg Lasers Imaging Retina.

[16] Kim JT, Lee DH, Joe SG, Kim JG, Yoon YH (2013). Changes in choroidal thickness in relation to the severity of retinopathy and macular edema in type 2 diabetic patients. Investig Ophthalmol Vis Sci..

[17] Adhi M, Brewer E, Waheed NK, Duker JS (2013). Analysis of morphological features and vascular layers of choroid in diabetic retinopathy using spectral-domain optical coherence tomography. JAMA Ophthalmol..

[18] Esmaeelpour M, Považay B, Hermann B (2011). Mapping choroidal and retinal thickness variation in type 2 diabetes using three-dimensional 1060-nm optical coherence tomography. Investig Opthalmol Vis Sci..

[19] Yiu G, Manjunath V, Chiu SJ, Farsiu S, Mahmoud TH (2014). Effect of anti-vascular endothelial growth factor therapy on choroidal thickness in diabetic macular edema. Am J Ophthalmol.

[20] Lauren Branchini M, Caio Regatieri P, Mehreen Adhi M (2017). Effect of intravitreous anti-vascular endothelial growth factor therapy on choroidal thickness in neovascular age-related macular degeneration using spectral-domain optical coherence tomography. JAMA Ophthalmol..

[21] Choi W, Waheed NK, Moult EM (2017). Ultrahigh speed swept source optical coherence tomography angiography of retinal and choroicapillaris alterations in diabetic patients with and without retinopathy. Retina..

[22] Nagaoka T, Kitaya N, Sugawara R (2004). Alteration of choroidal circulation in the foveal region in patients with type 2 diabetes. Br J Ophthalmol.

[23] Conti FF, Qin VL, Rodrigues EB, Sharma S, Rachitskaya AV, Ehlers JP, Singh RP (2018). Choriocapillaris and retinal vascular plexus density of diabetic eyes using split-spectrum amplitude decorrelation spectral domain optical coherence tomography angiography. Br J Ophthalmol.

[24] Takase N, Nozaki M, Kato A, Ozeki H, Yoshida M, Ogura Y (2015). Enlargement of foveal avascular zone in diabetic eyes evaluated by en face optical coherence tomography angiography. Retina..

[25] Kim K, Kim ES, Yu S-Y (2017). Optical coherence tomography angiography analysis of foveal microvascular changes and inner retinal layer thinning in patients with diabetes. Br J Ophthalmol..

[26] Campochiaro PA, Wykoff CC, Shapiro H, Rubio RG, Ehrlich JS (2014). Neutralization of vascular endothelial growth factor slows progression of retinal nonperfusion in patients with diabetic macular edema. Ophthalmology.

[27] Terui T, Kondo M, Sugita T (2011). Changes in areas of capillary nonperfusion after intravitreal injection of bevacizumab in eyes with branch retinal vein occlusion. Retina..

[28] Campochiaro PA, Bhisitkul RB, Shapiro H, Rubio RG (2013). Vascular endothelial growth factor promotes progressive retinal nonperfusion in patients with retinal vein occlusion. Ophthalmology.

[29] Sophie R, Hafiz G, Scott AW (2013). Long-term outcomes in ranibizumab-treated patients with retinal vein occlusion; the role of progression of retinal nonperfusion. Am J Ophthalmol..

[30] Feucht N, Schönbach EM, Lanzl I, Kotliar K, Lohmann CP, Maier M (2013). Changes in the foveal microstructure after intravitreal bevacizumab application in patients with retinal vascular disease. Clin Ophthalmol..

[31] Campochiaro PA, Clark WL, Boyer DS (2015). Intravitreal aflibercept for macular edema following branch retinal vein occlusion: the 24-week results of the VIBRANT study. Ophthalmology.

[32] Martin DF, Maguire MG (2015). Treatment choice for diabetic macular edema. N Engl J Med..

[33] Korobelnik JF, Do DV, Schmidt-Erfurth U (2014). Intravitreal aflibercept for diabetic macular edema. Ophthalmology.

[34] Diabetic Retinopathy Clinical Research Network (2007). The relationship between OCT-measured central retinal thickness and visual acuity in diabetic macular edema. Ophthalmology.

[35] Virgili G, Menchini F, Casazza G (2015). Optical coherence tomography (OCT) for detection of macular oedema in patients with diabetic retinopathy. Cochrane Database Syst Rev..

[36] Pedinielli A, Bonnin S, Sanharawi ME, Mané V (2017). Three different optical coherence tomography angiography measurement methods for assessing capillary density changes in diabetic retinopathy. Ophthalmic Surg Lasers Imaging Retina.

